# The epidemiology of hematogenous vertebral osteomyelitis: a cohort study in a tertiary care hospital

**DOI:** 10.1186/1471-2334-10-158

**Published:** 2010-06-07

**Authors:** Kavita P Bhavan, Jonas Marschall, Margaret A Olsen, Victoria J Fraser, Neill M Wright, David K Warren

**Affiliations:** 1 Division of Infectious Diseases, Washington University School of Medicine, St. Louis, MO, USA; 2 Department of Neurosurgery, Washington University School of Medicine, St. Louis, MO, USA

## Abstract

**Background:**

Vertebral osteomyelitis is a common manifestation of osteomyelitis in adults and associated with considerable morbidity. Limited data exist regarding hematogenous vertebral osteomyelitis. Our objective was to describe the epidemiology and management of hematogenous vertebral osteomyelitis.

**Methods:**

We performed a 2-year retrospective cohort study of adult patients with hematogenous vertebral osteomyelitis at a tertiary care hospital.

**Results:**

Seventy patients with hematogenous vertebral osteomyelitis were identified. The mean age was 59.7 years (±15.0) and 38 (54%) were male. Common comorbidities included diabetes (43%) and renal insufficiency (24%). Predisposing factors in the 30 days prior to admission included bacteremia (19%), skin/soft tissue infection (17%), and having an indwelling catheter (30%). Back pain was the most common symptom (87%). Seven (10%) patients presented with paraplegia. Among the 46 (66%) patients with a microbiological diagnosis, the most common organisms were methicillin-susceptible *S. aureus *[15 (33%) cases], and methicillin-resistant *S. aureus *[10 (22%)]. Among the 44 (63%) patients who had a diagnostic biopsy, open biopsy was more likely to result in pathogen recovery [14 (93%) of 15 with open biopsy vs. 14 (48%) of 29 with needle biopsy; p = 0.003]. Sixteen (23%) patients required surgical intervention for therapeutic purposes during admission.

**Conclusions:**

This is one of the largest series of hematogenous vertebral osteomyelitis. A microbiological diagnosis was made in only approximately two-thirds of cases. *S. aureus *was the most common causative organism, of which almost half the isolates were methicillin-resistant.

## Background

Vertebral osteomyelitis, also termed spondylodiscitis, is a common manifestation of osteomyelitis in adults [1,2]. It encompasses both infection due to a contiguous source, (i.e. post-surgical and trauma) and hematogenous spread [3]. Despite significant differences in pathogenesis, studies on vertebral osteomyelitis often do not distinguish between the specific infectious etiologies [4]. The prevalence of vertebral osteomyelitis appears to be increasing, though this may be due to a variety of factors including better diagnostic techniques, an aging and more immunocompromised population, increasing indwelling device use and intravenous drug use [5]. Hematogenous vertebral osteomyelitis is associated with significant morbidity including prolonged antimicrobial therapy, risk of recurrence, decreased functional status, and paralysis [4,6]. Previous studies of hematogenous vertebral osteomyelitis are limited by relatively small sample sizes [7] and were performed prior to the dramatic increase of MRSA in the community [8,9]. A more recent study suggests that the microbiology of this disease may be changing [10].

We performed a retrospective cohort study of patients with hematogenous vertebral osteomyelitis in an effort to gain a better understanding of the current clinical presentation, management and treatment of this disease entity.

## Methods

### Setting

Barnes-Jewish Hospital (BJH), a 1250-bed teaching hospital, is the largest hospital in Missouri, with a referral base that includes the Saint Louis metropolitan area, eastern Missouri and western Illinois.

### Study design

We performed a two-year, retrospective cohort study of patients admitted to Barnes-Jewish Hospital between January 1, 2004 and December 31, 2005, and diagnosed with hematogenous vertebral osteomyelitis. We utilized the BJH Medical Informatics database to identify all discharges with ICD-9-CM codes for vertebral osteomyelitis, spondylodiscitis, disk infection, and epidural abscess [*epidural abscess *(324.1 and 324.9), *osteomyelitis of the spine or unspecified site *(730.28, 730.08, 730.2, 730.00), and *discitis *(722.90, 722.91, 722.92, 722.93)]. Discharges containing these ICD-9-CM codes were then reviewed to determine if they met study criteria defined below. The study was approved by the Washington University Human Research Protection Office.

### Inclusion and exclusion criteria

Patients ≥ 18 years old admitted to the hospital who presented with or developed hematogenous vertebral osteomyelitis as defined by characteristic radiographical changes including decreased signal intensity in the vertebral body and disk and loss of endplate definition on T1 weighted images and increased signal intensity of the disk and vertebral body on T2 weighted images. We excluded patients with contiguous osteomyelitis due to decubitus ulcers, trauma, and surgical site infection (defined as infection within one year of spinal surgery). Stage I decubitus ulcers were not considered a reason for exclusion. Patients with incomplete medical records (n = 4) were also excluded.

### Data collection

We reviewed medical records of all the patients who met study criteria. Medical records were reviewed for demographic information, comorbidities, antibiotic history, presenting symptoms, vital signs and physical examination findings, diagnostic procedures, microbiology, and medical and surgical treatment. Laboratory values and temperature data within twenty-four hours of admission were collected.

### Definitions

Renal insufficiency was defined as serum creatinine of ≥ 2 mg/dL. Active malignancy was defined as having received chemotherapy or radiation therapy for cancer in the previous six months. For coagulase-negative staphylococci or other possible skin contaminants to be considered a true pathogen, they had to be isolated from a sterilely obtained bone biopsy and/or two or more blood cultures drawn on separate occasions.

### Statistical analysis

Data entry was performed using Microsoft Access and Excel (Microsoft Corp., Redmond, WA) and data analysis was performed using SPSS 14 (SPSS Inc., Chicago, IL). Univariate comparisons among categorical variables were performed using the χ^2 ^test or Fisher's exact test, as appropriate. Comparisons among continuous independent variables were performed using Student's t test or Mann Whitney U test, as appropriate. A two-sided *p *value of < 0.05 was considered significant.

## Results

### Patient characteristics and clinical presentation

Of the 272 patients with ICD-9-CM codes of interest, 70 were identified as having hematogenous vertebral osteomyelitis and met study criteria (Table 1). The mean age was 59.7 years (standard deviation ±15, range 44-74) and 38 patients were male (54.3%). Chronic renal insufficiency and diabetes mellitus were the leading comorbidities in our patient cohort. Thirteen (18.6%) patients received chronic hemodialysis therapy. Seven patients (10.0%) had received systemic corticosteroids in the recent past, of which five had received treatment in the last month.

**Table 1:** Patient characteristics and clinical presentation in 70 cases of hematogenous vertebral osteomyelitis

Variable	n (%)
Age (mean, ± standard deviation), years	59.7 (±15.0)

Male gender	38 (54%)

Caucasian race	44 (63%)

Body mass index (median, range), kg/m^2^	27.2 (13.4-80.6)

Transfer from outside hospital	31 (44%)

**Comorbidities**	

Diabetes mellitus	30 (43%)

Degenerative joint disease	30 (43%)

Renal insufficiency (serum-creatinine > 2 mg/dl)	17 (24%)

History of spinal injury	12 (17%)

History of spinal surgery (>1 year ago)	10 (14%)

HIV infection	1 (1%)

Malignancy	1 (1%)

Solid organ transplant/Bone marrow transplant	1 (1%)

**Procedures or infections in prior month**	

Urinary tract infection	14 (20%)

Indwelling catheter	21 (30%)

Bacteremia	13 (19%)

Skin or soft tissue infection	12 (17%)

Pneumonia	7 (10%)

Colonoscopy	2 (3%)

Dental procedure	2 (3%)

**Reported symptoms**	
Back pain	61 (87%)

Weakness	39 (56%)

Fever	32 (46%)

Radicular pain	24 (34%)

Sensory loss	20 (29%)

Weight loss	14 (20%)

Stool or urine incontinence	9 (13%)

**Physical findings**	

Decreased deep tendon reflexes	46 (70%)

Impaired motor strength	40 (60%)

Sensory deficit	29 (44%)

Decreased rectal tone*	10 (14%)

Positive Babinski's sign	9 (13%)

NOTE. Data are presented as number (% of total) of cases, unless otherwise indicated. * Data available for 33 patients

Forty-six (65.7%) patients had a documented infection at another site within the thirty days prior to admission. Thirty-one (44.3%) patients had been transferred from an outside hospital to our institution. Compared to patients admitted directly to our hospital, patients transferred from an outside hospital were more likely found to have a history of documented pneumonia [6 (8.6%) transfer vs. 1 (1.4%) non-transfer, p = 0.03] or bacteremia [10 (14.3%) transfer vs. 3 (4.3%) non-transfer, p = 0.01] in the thirty days prior to admission. In terms of other predisposing factors and signs, such as neurological deficits/paralysis, there were no statistical differences observed between the two groups.

The most frequently reported symptoms on admission were back pain in 61 (87.1%) patients, weakness in 39 (55.7%), and fever in 32 (45.7%). Prior to admission, the median duration of back pain was a median of 17.5 days (range 1-365 days). The median duration of weakness was 14 days (range 1-120 days). The median duration of fever was 6.5 days (range 1-30 days). Twenty-eight (40%) patients in our cohort presented with a normal white blood cell count. Of patients who had laboratory tests for ESR and CRP (n = 58 and n = 40, respectively) performed, only one patient had an ESR within normal limit and two had normal CRP levels (Table 2).

**Table 2:** Diagnostic workup, treatment, and outcomes of 70 patients with hematogenous vertebral osteomyelitis

Variable	n (%)
Laboratory data*	

WBC count within first 24 hours of admission (median, range), K/mm^3^	11.5 (3.3-42.7)

ESR (median, range), mm/h	93.5 (8-140)

CRP (median, range), mg/dL	88.7 (0.4-472)

Temperature within first 24 hours (median, range), °C	37.4 (36.6-40.0)

**Diagnostic work-up**	

Blood culture on admission	60 (86%)

Needle biopsy	29 (41%)

Open biopsy	15 (21%)

**Treatment**	

Any surgery performed	16 (23%)

Laminectomy/Discectomy	10 (14%)

Laminectomy/Fusion	5 (7%)

Corpectomy	1 (1%)

**Outcomes**	

Paralysis developed during admission	1 (1%)

Median hospital length of stay (days, range)	12 (3-59)

Discharge location	

Home or home health	25 (36%)

SNF/LTCF/Rehabilitation	41 (59%)

In-hospital mortality	3 (4%)

NOTE. Data are presented as number (% of total) of cases, unless otherwise indicated. * denotes maximum values. WBC = white blood cell count (normal range 3.8-9.8 per BJH laboratory medicine). CRP = C-reactive protein; data available for 40 patients (high risk => 3 mg/L per BJH laboratory medicine). ESR = erythrocyte sedimentation rate; data available for 58 patients (normal 0-35 per BJH laboratory medicine). SNF = skilled nursing facility. LTCF = long term care facility.

A complete neurological exam was documented for 66 patients. Nine patients (12.9%) in our cohort were noted to have a normal neurological exam on admission (Table1). Twenty patients (33.9%) had one neurological deficit, sixteen (27.1%) had two deficits, and twenty-three patients (39.0%) had three or more documented deficits.

### Diagnostic evaluation

All 70 patients underwent radiological evaluation. The most common radiological diagnoses were discitis (75.7%), vertebral osteomyelitis (67.1%), epidural abscess (35.7%), and paraspinal abscess (21.4%). The lumbar spine was the most common location of infection (47.1%), followed by the thoracic (28.6%) and cervical spine (24.3%). In addition to radiographic and microbiological findings, in 19 (27.1%) cases pathological findings confirmed the diagnosis of vertebral osteomyelitis.

The Infectious Diseases service was consulted in 65 (92.9%) of cases to guide diagnostic procedures and therapy. Neurosurgery was consulted in 54 (77.1%), and orthopedic surgery in 11 (15.7%) cases.

Forty-six (66%) patients had a microbiological diagnosis made by bone biopsy and/or positive blood culture (Table 3). Eighteen patients (26%) were diagnosed by bone biopsy, 18 (26%) by blood culture, and an additional 10 (14%) cases had positive cultures both from bone and blood specimens. Two out of 70 patients had polymicrobial infections. In cases with a microbiological diagnosis, *Staphylococcus aureus *was isolated 54% of the time, with 40% of these cases being MRSA. Patients with a confirmed microbiological diagnosis by either bone or blood culture were more likely to have an elevated temperature, WBC and CRP level (Table 4).

**Table 3:** Microbiology of 70 cases of hematogenous vertebral osteomyelitis from blood cultures and/or bone biopsies

Organism detected	n (%)
MSSA	15 (21)

MRSA	10 (14)

Coagulase-negative staphylococci	7 (10)

*Streptococcus spp.*	4 (6)

*E. coli*	3 (4)

*P. aeruginosa*	2 (3)

*E. faecalis*	2 (3)

*A. baumannii*	1 (1)

*E. cloacae*	1 (1)

*Propionibacterium sp.*	1 (1)

No organism recovered	24 (34)

NOTE. Blood and/or bone cultures were performed in all 70 patients. MSSA = methicillin-susceptible *Staphylococcus aureus*. MRSA = methicillin-resistant *S. aureus*. There were five cases of coagulase-negative staphylococci diagnosed with blood cultures, and two diagnosed by bone biopsies and blood cultures. The single *Propionibacterium sp*. infection was diagnosed by bone biopsy. There were two polymicrobial infections: one *Staphylococcus aureus *infection together with unidentifiable Gram-negative bacilli, and one coagulase-negative staphylococcal and *Corynebacterium sp*. infection.

**Table 4:** Comparison of hematogenous vertebral osteomyelitis patients based on presence of a microbiological diagnosis

Variable	Microbiological diagnosis n = 46	No microbiological diagnosis n = 24	p value
Mean age (±SD)	57.5 (±15.5)	63.9 (±13.3)	0.08
Diabetes mellitus	18 (39.1%)	12 (50.0%)	0.4
Renal insufficiency	12 (26.1%)	5 (20.8%)	0.6
Bacteremia, last 30 days	7 (15.2%)	6 (25.0%)	0.3
History of indwelling catheter, last 30 days	12 (26.1%)	9 (37.5%)	0.3
Maximum temperature at admission (±SD), °C	38.0 (±0.8)	37.3 (±0.7)	<0.001
Maximum WBC at admission (±SD), K/m m^3^	15.1 (±9.2)	9.4 (±4.8)	0.003
CRP at admission (±SD), mg/dL	132.2 (±107.4)	75.3(±112.2)	0.04
ESR at admission (±SD), mm/h	89.5 (32.4%)	83.6 (36.6%)	0.6

NOTE. SD = standard deviation. WBC = white blood cell count. CRP = C-reactive protein. ESR = erythrocyte sedimentation rate.

Forty-four (63%) patients underwent needle or open surgical biopsy of the spine as diagnostic evaluation (Table 2). The median time from admission to bone biopsy was 2.5 days (range 0-69). Among the patients who had a diagnostic biopsy, open biopsy was more likely to result in pathogen recovery [14 (93%) of 15 open biopsies vs. 14 (48%) of 29 needle biopsies; p = 0.003]. Sixteen of 44 patients had culture-negative bone biopsies. In 18 cases, blood cultures were used to guide treatment and of these patients 14 never underwent a bone biopsy.

### Treatment and outcomes

Sixteen (23%) patients had a surgical procedure performed during admission for therapeutic purposes as indicated by neurological compromise, mechanical instability, etc. The most common surgical intervention was laminectomy/discectomy in 10 (14%) patients, followed by laminectomy/vertebral fusion in 5 (7%) patients and 1 patient (1%) underwent a corpectomy procedure.

Fifty-five patients (78.6%) received antibiotic therapy within 48 hours of admission (range 0-5 days). The most frequently planned total duration of antibiotic therapy at discharge was six weeks, and 11 (15.7%) patients were scheduled to complete longer courses (7-12 weeks) of antibiotics. When we examined patients with known positive blood cultures from an outside hospital prior to transfer in combination with patients who had positive blood or bone cultures at our hospital, we found that fifty (71.4%) patients received treatment matching microbiology susceptibility patterns for a recovered pathogen. Adequate empirical treatment was administered to an additional 17 (24.3%) patients without a microbiological diagnosis and consisted of a combination of vancomycin and either a third generation cephalosporin or a carbapenem.

A large number of patients presented with neurological deficits, among whom six had a diagnosis of paralysis at time of admission. Only one patient developed paralysis during hospitalization, and all-cause mortality was low (Table 2). The median length of hospital stay was 12 days (range 3-59 days). The majority of patients were discharged to a skilled nursing care/long term care facility.

## Discussion

Our cohort represents one of the largest, single-center studies of hematogenous vertebral osteomyelitis. Previous studies have often included surgical site infections and other contiguous infections of the spine when describing the epidemiology of vertebral osteomyelitis. Hematogenous vertebral osteomyelitis as a separate entity, however, has infrequently been studied [4,7,11,12]. These previous studies failed to clearly define exclusion criteria regarding the presence of sacral decubitus ulcers, which can be a cause of vertebral osteomyelitis [13]. In our study, patients were excluded on the basis of having decubitus ulcers stage II or greater. These patients were the second most common subset of vertebral osteomyelitis in our institution, after surgical site infections of the spine.

In terms of patient characteristics, we found chronic renal insufficiency and diabetes mellitus to be the most frequent comorbidities among patients with hematogenous vertebral osteomyelitis in our study population, similar to what has been described in previous papers [4,7,14]. The frequency of intravenous drug abuse as a potential predisposing factor, however, was less common as only 3 (4.3%) patients with history of IV drug abuse admitted to drug use in the month prior to admission compared to the 25% frequency reported by Hadjipavlou [7]. The pathogenesis of hematogenous vertebral osteomyelitis implies the presence of bacteremia. Forty percent of patients had bacteremia during the hospital stay and 19% of patients had a history of bacteremia in the month prior to admission. Of the 28 patients with a positive blood culture during their hospital stay, four patients had a history of bacteremia in the month prior to admission. Our findings are consistent with previously published reports stating that blood cultures provide microbiological diagnosis in 20-59% of cases [15]. Other potential sources of spinal infection in the absence of detected bacteremia in the study cohort included urinary tract infections, skin and soft tissue infections, and pneumonia, as reported previously [6].

The most frequently reported symptoms at presentation were back pain, weakness, and fever. Unlike previous studies which described symptoms for greater than three months prior to diagnosis [12], we report a shorter duration of symptoms. This may be due to several factors including increased virulence of organisms, improved diagnosis and imaging and possible misreporting in our patient population, or may be related to increased access to healthcare in an urban tertiary care setting.

Obtaining a microbiological diagnosis is crucial given the need for prolonged antibiotic treatment and the potential for increased cost, toxicity, and other adverse events associated with broad-spectrum antibiotic use. While biopsy is an important part of the diagnostic workup of vertebral osteomyelitis, the microbiological yield was only 40-60% in previous studies [7,16], similar to the 64% yield (28/44 bone cultures) in our study. Several mechanisms have been cited in the literature to explain non-diagnostic biopsies, e.g. antibiotic administration prior to biopsy, poor sampling technique, and the resolution of infection [7]. In 26% (18 out of 70) of our cases, blood culture results alone were used to guide therapy in the absence of positive results from bone biopsy.

Longitudinal data from the 1990's show *S. aureus *as an increasing cause of hematogenous vertebral osteomyelitis [17]. In contrast to previous studies [11], we noticed a higher percentage of methicillin-resistant *Staphylococcus aureus*. We observed 45% of our *S. aureus *isolates to be methicillin-resistant. This corresponds to an increase in methicillin-resistant *Staphylococcus aureus *bacteremia over the last decade [18]. This, along with our finding that 15% (7 of 46) of microbiologically diagnosed infections were due to coagulase-negative *Staphylococcus*, underscores the need for including antimicrobial coverage against methicillin-resistant Gram-positive bacteria as part of empirical regimens. Recent evidence of higher vancomycin MIC levels in MRSA isolates must be taken into account to assure adequate dosing of vancomycin to overcome these higher MIC levels in circulating strains [19].

There are a few limitations to this study. We used a retrospective design and therefore may have missed undocumented predisposing factors. ICD-9-CM codes were used to identify cases and we may have missed cases which were misclassified as a result. The data is derived from one tertiary care center, which may limit generalizability. Long-term outcomes of hematogenous vertebral osteomyelitis, for example functional status after discharge, and treatment outcomes could not be obtained on this cohort. Among the strengths of this study are the stringent use of exclusion criteria to define our patient population and the size of the study. This is one of the largest single-center cohorts of hematogenous vertebral osteomyelitis reported to date.

## Conclusions

In summary, we report descriptive data from a retrospective cohort of hematogenous vertebral osteomyelitis. We observed that only two-thirds of patients had a microbiological diagnosis confirmed. MRSA was isolated in a substantial proportion of cases and more frequently than what has been previously published. This has important implications for empirical treatment in the absence of specific microbiological diagnosis, which should include antimicrobials with activity against MRSA. Typical symptoms of infection were often absent in our cohort. Signs of fever and leukocytosis were not reliable in establishing the diagnosis of hematogenous vertebral osteomyelitis, and patients often only presented with symptoms of chronic back pain. The lack of signs which are usually indicative of infection may confuse the clinical picture and cause delays in diagnosis. Delays in diagnosis are associated with adverse outcomes [6]. It is important for physicians to maintain a heightened level of suspicion for osteomyelitis in the setting of persistent back pain and any changes in neurological exam to initiate appropriate laboratory and radiologic evaluations in order to make a timely diagnosis.

## Competing interests

None of the following authors has a conflict of interest (KP Bhavan, J Marschall, MA Olsen, NM Wright). DK Warren is a Consultant for 3 M Healthcare, Novabay Pharmaceuticals, and Cardinal Health, and receives research funding from Sage Products, Inc., Cubist Pharmaceuticals and 3 M Healthcare. VJ Fraser has been a Consultant for Steris and Verimetrix, and Member of the Speakers Bureau for Pfizer, Merck, and Cubist Pharmaceuticals in the past three years.

## Authors' contributions

DKW and KPB devised the study; MAO generated the query based on ICD-9-CM codes; NMW and VJF assisted in study design; KPB and JM collected and analyzed the data, and wrote the manuscript; all authors critically reviewed the final draft of the manuscript.

## Pre-publication history

The pre-publication history for this paper can be accessed here:

http://www.biomedcentral.com/1471-2334/10/158/prepub
